# The Curability and Treatment of Pulmonary Phthisis

**Published:** 1885-03

**Authors:** 


### Notes on Books.

*The Curability and Treatment of Pulmonary Phthisis.* By S. JACCOUD, Professor of Med. Pathology to the Faculty of Paris, &c. Translated by MONTAGU LUBBOCK, M.D. London: KEGAN PAUL, TRENCH, & Co. 1885.

This book, written by one of the best authorities on the subject, contains the substance of lectures delivered in December, 1880, and January, 1881, before the discovery of the tubercle bacillus by Dr. Robert Koch in 1882. At the meeting of the International Congress in Copenhagen, in 1884, it was asserted by Dr. Jaccoud, in elegant and forcible language, that the discovery of the bacillus had been absolutely sterile in its effects upon the treatment of phthisis, and that the discovery was of no value in a therapeutic sense. Accordingly, one would not expect to find in this book any of the later methods of treatment directed towards the destruction of the bacillus, and a perusal of the 407 pages of matter contained therein enables the reader to perceive on what foundation Dr. Jaccoud's statement rests. It might be supposed that an account of the treatment of phthisis in which the bacillus is left out of consideration would be quite out of date; but the author strongly held to the opinion that tuberculosis is an infectious disease, that the infective agent passes in some way from person to person, and that auto-infection is possible, the virus being disseminated through the body from pre-existing caseous or tuberculous deposits. The nature of the agent was quite unknown to him; nor could he describe its conditions of life, as can now be done. Malnutrition was believed to be the chief factor in the liability to tuberculosis; and the actual production of tubercle, whether it be an exudation or cellular formation, was regarded as due to an irritative process termed "phymatogenous irritation," or to actual inflammation of the lung.

The discovery of the bacillus has only confirmed the truth of such views as these. The most ardent advocate of the causal relationship of the bacillus to the disease cannot set aside the questions of malnutrition and the resisting power of healthy tissues to oppose the germination and reproduction of bacilli. The opinions of the author—built up on a very large experience, established on so advanced a pathological basis, described and illustrated in forcible language, and containing much that is new in the details of treatment—cannot fail to be of interest. Further, another fact gives the book an especial interest: the author holds strongly "that phthisis is curable in all its stages; this is the prolific notion which presides over the whole history of the disease, and which should increasingly inspire and direct all medical action. The incurability proclaimed by Laennec and his followers is disproved by pathological anatomy and clinical observation. None should therefore allow themselves to be influenced by such condemnation, which is but a historical souvenir. When the existence of tubercles in the lung is recognized, it should not be inferred from that moment that he who has them is doomed to death in consequence of their presence. . . . The enemy can be conquered: this is the idea which should engender and sustain every effort" (p. 28).

It will be naturally asked whether the author has any special methods of treatment by which his results have been obtained, and it will be of interest to elucidate any special features by which his treatment differs from that which is generally employed. In two chapters, comprising 58 pages, he lays great stress on the *prophylactic* treatment, which includes not only all the usual hygienic measures, but comprises also hydro-therapeutic, and aëro-therapeutic or pneumatic treatment, by means of compressed air. Concerning this last method he says: "In short, continued maintenance of the lesions in a stationary condition, more or less marked diminution in their extent, or even their complete disappearance, may be expected if the practice of aëro-therapeutics be properly carried out." He advises that a good red wine should form part of the diet, even in the case of those persons who have not been accustomed to take it; and in the morning and afternoon, between the two meals, a quantity of milk, increased gradually to nearly a quart (a litre). On this latter point he says: "If possible, the milk should be drunk in the cowshed at the time of being drawn;" and further: "My conviction is so strong that I give this advice" (breathing the air of the cowshed for one hour daily) "even to patients who are unwilling or unable to take milk." Koumiss is well spoken of as easy of digestion, and combining the nutritive qualities of the milk with the stimulating effects of the alcoholic beverages. As regards *medicinal* treatment, he places cod liver oil first, as the agent which seems to be the most powerful in its therapeutic results; but it should be given in larger doses than are commonly employed, as no advantage will be gained by the daily administration of one or two teaspoonfuls. When two tablespoonfuls can be taken daily, there will be but little difficulty in increasing the amount to four or even six tablespoonfuls; and this is the least quantity considered, in the author's opinion, to be of real service. He would limit the quantity only by the capacity of the patient to digest it. Many patients can take from seven to ten ounces daily, and the remedy should be given in as large a quantity as can be borne, three or four ounces being considered the minimum dose necessary. He considers the presence of pyrexia to be an absolute contra-indication to cod liver oil, which should then be replaced by glycerine, which is well digested even when pyrexia exists. One or other of these two agents may be permanently administered, the one occasionally taking the place of the other; and the glycerine is found to supply the place of the cod liver oil to an extent which is really beneficial. The dose of glycerine given is three to four tablespoonfuls daily, combined with a drop of essence of mint and two drachms of brandy or rum, to modify the insipidness and to promote digestion.

Arsenious acid, in granules of one milligramme, is also given; two granules daily at first, gradually increased up to six, eight, or ten. The association of arsenic with cod liver oil or glycerine is believed to be the most sure means of obtaining that organic repair which is needed, and "such treatment is not only sure, but infallible in this sense, that after a time, which varies from five to six weeks, a positive improvement is always found to occur in the general condition, the appearance, the strength, and weight of the patient" (p. 145).

At the same time that hygienic, mechanical, and medicinal means of treatment are directed to the general condition of the patient, certain fundamental indications furnished by the local lesion should not be overlooked, and counter-irritation should be not only adopted, but continued with unswerving perseverance. The form of counter-irritation adopted is the Vienna paste, which is applied in three places beneath the clavicle on one or both sides, according to the seat of the lesions; croton oil, tincture of iodine, and other applications, are also employed.

The tendency of the disease to induce pulmonary catarrh is to be combated by means of balsams, tar, or turpentine, taken internally or in the form of inhalation. Creosote is believed to be by far the best of this class of remedies, as it "acts more rapidly and with greater certainty; it diminishes the expectoration, and by its effect upon the bronchial tubes prevents any extension of the catarrhal lesions, thus notably reducing the extent of the pulmonary changes. Nor is this all, since creosote seems also to have some effect upon the fundamental lesions themselves, and to promote the sclerotic change, by means of which recovery is found to occur." It should be given in small doses at first—three minims daily, and increased one minim in each week until as much as five or even six minims are given daily. This dose should never be exceeded, and is but rarely reached, since a small dose is effectual, and does not expose the patient to the danger of gastralgia: it may be combined with the cod liver oil, the three minims of creosote being mixed with the daily dose of oil, and one drop of essence of peppermint added. The following combination is also found to be pleasant and well tolerated: glycerine, twelve drachms; cognac or rum, three drachms; creosote, three to six minims; essence of peppermint, one minim: this mixture being the quantity for one day, given in divided doses.

To subdue pyrexia, the hydric bromate of quinine, in doses of about twenty-two grains, is administered; and if gastric troubles contraindicate it, the drug is given hypodermically. Salicylic acid is, however, generally considered preferable, as it combines a more powerful antiseptic action with its antipyretic effects; thirty grains are given on the first day, and twenty or fifteen grains on the second and third days; the most convenient form of administration is that of tablets of seven grains, given four to seven within one hour, a large glass of water containing two or three teaspoonfuls of brandy being taken with each tablet.

The inhalation of carbolic acid and solution of benzoate of soda are believed to be of service when active excavation is going on; but when the cavities are dry and of fixed size inhalations do not appear to be of service.

The treatment of the various digestive symptoms—vomiting, diarrhœa, &c.—does not call for special comment, nor do the measures used for checking hæmoptysis.

The four remaining chapters of the book are a voluminous account of treatment by mineral waters and climates. The personal acquaintance with the various places mentioned which the author claims gives him a right to speak with authority in this part of his subject, as he does on all other topics throughout the volume: the personality of the writer is a striking feature through all its pages. One would like to know on what amount of evidence some of the conclusions are founded; but the phrase, "in my opinion," is one which teems on almost every page, and which one must accept as the authoritative dictum of one who feels that he is in all points master of his subject, and has much to teach.

There is, however, one point on which the author is silent, and ere long a second edition will be necessary, in order that the newer methods of treatment, founded on the specific nature of the disease and its parasitic origin, may be fully described, and the results detailed. On this subject it is yet premature to dictate, as the various methods are still *sub judice*. Some of these have already obtained results superior to those recorded previously; the internal administration of iodoform, the method of continuous inhalation advocated by Burney Yeo and Solis Cohen, and the use of intra-pulmonary injections, hold out a hope that the phthisical patients of the future will have a better prospect, not only of temporary relief but of permanent recovery, than even the encouraging programme held out to them by Dr. Jaccoud.

